# Effects of clusterin over-expression on metastatic progression and therapy in breast cancer

**DOI:** 10.1186/1471-2407-10-107

**Published:** 2010-03-22

**Authors:** Louise Flanagan, Lorna Whyte, Namita Chatterjee, Martin Tenniswood

**Affiliations:** 1 Department of Biological Sciences, University of Notre Dame, Notre Dame, IN 46556, USA; 2 Cancer Research Center and Department of Biomedical Sciences, School of Public Health, State University of New York at Albany, Rensselaer, NY 12144, USA

## Abstract

**Background:**

Clusterin is a secreted glycoprotein that is upregulated in a variety of cell lines in response to stress, and enhances cell survival. A second nuclear isoform of clusterin that is associated with cell death has also been identified. The aim of this study was to determine the role(s) of the secretory isoform in breast tumor progression and metastasis.

**Methods:**

To investigate the role of secretory clusterin in the biology of breast cancer tumor growth and resistance to therapy we have engineered an MCF-7 cell line (MCF-7CLU) that over-expresses clusterin. We have measured the *in vitro *effects of clusterin over-expression on cell cycle, cell death, and sensitivity to TNFalpha and tamoxifen. Using an orthotopic model of breast cancer, we have also determined the effects of over-expression of clusterin on tumor growth and metastatic progression.

**Results:**

In vitro, over-expression of secretory clusterin alters the cell cycle kinetics and decreases the rate of cell death, resulting in the enhancement of cell growth. Over-expression of secretory clusterin also blocks the TNFalpha-mediated induction of p21 and abrogates the cleavage of Bax to t-Bax, rendering the MCF-7CLU cells significantly more resistant to the cytokine than the parental cells. Orthotopic primary tumors derived from MCF-7CLU cells grow significantly more rapidly than tumors derived from parental MCF-7 cells and, unlike the parental cells, metastasize frequently to the lungs.

**Conclusions:**

These data suggest that secretory clusterin, which is frequently up-regulated in breast cancers by common therapies, including anti-estrogens, may play a significant role in tumor growth, metastatic progression and subsequent drug resistance in surviving cells.

## Background

The development of a metastatic, hormone-independent and drug resistant phenotype is responsible for a high percentage of treatment failures among breast cancer patients. Therefore, understanding the molecular mechanisms of metastasis is crucial for the design and effective use of novel therapeutic strategies to combat tumor progression [1-3]. Whether the phenotypes of drug resistance, hormone independence and invasion are linked genotypically, or whether they involve independent genetic or epigenetics processes, remains to be determined. Clusterin is a secreted heterodimeric 70-80 kDa glycoprotein composed of alpha and beta chains linked by five inter-chain disulfide bonds [4,5]. The glycoprotein is induced during apoptosis in hormone dependent tissues including the prostate and mammary gland [6,7], as well as many other tissues in response to stress [8-11], although it is not clear whether the protein plays a pivotal mechanistic role in either cell death or cell survival since it is also expressed constitutively in many tissues [9,11].

In MCF-7 breast cancer cells, the biogenesis of clusterin is significantly altered during tamoxifen-induced apoptosis, and a new isoform of the protein appears in the nucleus [12]. While the details of the changes in the intracellular trafficking that produce this new isoform have not been fully elucidated, it is clear that the nuclear isoform is not cleaved to form the alpha and beta chains and is not glycosylated, even though the protein appears to retain its disulfide linkages [12]. Increases in a nuclear form of clusterin in response to radiation [13], heat shock [14] and other stress inducers [15-17] have also been described although there is no clear consensus on the biogenesis or structure of the nuclear protein or its function. The nuclear isoform produced by radiation is known to bind to Ku70/80, inhibiting non-homologous end joining (NHEJ) DNA repair during apoptosis [18]. While the synthesis of clusterin and the appearance of the nuclear isoform is clearly upregulated during apoptosis, the role of the secretory protein in either cell death or cell survival has not been clearly delineated.

The physiological function of secreted the glycosylated isoform is not fully understood, but it most likely serves as a extracellular chaperone [19], although the structure-function relationship remains to be firmly established [4,20]. The protein is also known to bind to cholesterol and other hydrophobic ligands [21], giving rise to the suggestion that it is responsible for the retrograde transport of cholesterol and proteins in redundant membranes produced by apoptotic cells to the liver. Pathological or engineered over-expression of clusterin in various cell types confers resistance to the induction of apoptosis by several cytokines including TGFbeta, and TNFalpha [22-24] and promotes tumor progression in the prostate [25,26]. Thus, while the protein is clearly expressed in cells that are destined to undergo apoptosis, the observation that high levels of clusterin expression is also seen in surviving cells suggests that it may play a role in cell survival. Elevated clusterin expression generally correlates with tumor grade in prostate cancer [27-29], although at least one report has shown that the level of clusterin mRNA in prostate cancer is lower than in paired benign tissue from the organ [30]. Clusterin is currently being targeted using anti-sense RNA approaches in clinical trials for prostate cancer disease [31]. In breast cancer, two small retrospective studies has shown an association with elevated clusterin expression and large tumor size, estrogen and progesterone receptor status and with the progression from the primary carcinoma to metastatic carcinoma in lymph nodes in breast cancer [32,33]. The studies described here are designed to investigate whether secretory clusterin expression plays a causative role in the progression of human breast carcinoma.

## Methods

### Cell culture

MCF-7 (American Type Culture Collection, Rockville, MD), MCF-7CLU and SUM-159PT (University of Michigan Human Breast Cell/Tissue Bank, Ann Arbor, MI) were cultured in alpha-MEM medium (Life Technologies Inc, Gaithersburg, MD) containing 25 mM HEPES and 5% fetal bovine serum (ATLAS Biologicals, Fort Collins, CO). Cells were routinely passaged every 4-5 days. For cell growth and morphological assays, MCF-7 and MCF-7CLU cells were plated at either 10,000 or 20,000 cells/well in 24-well plates and were treated with 1, 5 or 10 micromolar tamoxifen (Sigma, St. Louis, MO), or 1, 5 or 10 ng/ml TNFalpha(Sigma) or ethanol vehicle for the indicated times, starting two days after plating.

### Over-expression of clusterin

MCF-7 cells were engineered to over-express the glycosylated clusterin isoform using Gateway Technology (Invitrogen Corporation, Carlsbad, California) according to the manufacturer's protocol. Briefly, Gateway's attB1 and attB2 recombination sites were added to the 5' and 3' ends respectively of human clusterin cDNA [34] by PCR amplification using primers containing the attB sites. Using Gateway Technology, which allows for the transfer of DNA among different vectors based on lambda-phage recombination, the clusterin cDNA was inserted into the pDONOR 201 plasmid to generate the entry clone. This vector was subsequently transferred into the pcDNA-DEST47 destination vector to create the expression clone pEXP-CLU which allows for constitutive gene expression under the control of a CMV promoter. A second construct was also engineered in which the stop codon was altered by a single base pair mutation of TGA to GGA yielding a glycine residue substitution. This alteration was generated to allow for expression of a C-terminal GFP-tagged clusterin (pEXP-CLUGFP). The construct mutations and correct transfer into pcDNA-DEST47 were confirmed in all cases by automated DNA sequencing.

The expression constructs (pEXP-CLU and pEXP-CLUGFP) were independently transfected into MCF-7 cells using Lipofectamine 2000 (GibcoBRL) according to the manufacturer's direction and MCF-7CLU cells were selected for by growth in appropriate media containing neomycin (BRL, Gaithersberg, MD).

Cell growth was determined by crystal violet assays. For quantitation of total adherent cell number, cells were fixed with 1% gluteraldehyde (Fisher Scientific, Pittsburgh, PA) for 15 min, incubated with 0.1% crystal violet (Fisher Scientific) for 30 min, destained with H_2_O, and solubilized with 0.2% Triton X-100 (Sigma). Absorbance at 590 nm, which is proportional to total adherent cell number, was determined on a microplate reader.

### PCR analysis

DNA was isolated from MCF-7 and MCF-7CLUGFP cells using a commercially available assay according to the manufacturer's protocol (Qiagen, Valencia, CA). Primers were designed that spanned the CLUGFP gene sequence and the pcDNA-DEST vector. DNA was amplified directly using primers specific for the CLU-pcDNA-DEST vector to generate a product of 380 bp. For MCF-7CLU-pcDNA-DEST47 the primers were as follows:

5'primer: 5'-TCC CAC ACT TCT GAC TCG GAC GTT C-3'

3'primer: 5'-GCA TCA CCT TCA CCC TCT CCA CTG A-3'

The amplification conditions were as follows: A denaturation step for 5 min at 95°C, followed by 45 sec at 95°C, 45 sec at 64°C and 45 sec at 72°C for 30 cycles, followed by 15 min at 72°C. 20 microliters of the PCR reaction was analyzed on a 1.5% agarose gel.

### Fluorescence microscopy

MCF-7 and MCF-7CLU cells were plated at 10,000 cells/well onto poly-L-lysine coated Lab-Tek II chamber slides (Fisher Scientific) and fixed in 4% formaldehyde after 72 h growth. Cells were analyzed by phase and fluorescence microscopy and photographed using an Olympus AX70 microscope equipped with a Spot RT digital camera.

### Flow cytometry

MCF-7 and MCF-7CLU cells were plated at a density of 8 × 10^5 ^cells/150-mm flask. 48 h following plating, cells were treated with 1, 2.5 or 5 micromolar tamoxifen or 1, 2.5 or 5 ng/ml TNFalpha or ethanol vehicle for the indicated times. For analysis of cell cycle kinetics, MCF-7 and MCF-7CLU cells were harvested by trypsinization, pooled with floating cells, pelleted by centrifugation (500 × g, 5 min, 4°C), fixed and permeabilized with 90% ethanol at -20°C. Cells were stained with 5 micrograms/ml propidium iodide (Sigma) containing RNase A (Roche Molecular Biochemicals, Mannheim, Germany). For analysis of DNA fragmentation, MCF-7 and MCF-7CLU cells were harvested by trypsinization at the indicated times, collected by centrifugation, fixed in 2% formaldehyde in PBS, and permeabilized in 70% ethanol at -20°C. DNA strand breaks in cells undergoing apoptosis were indirectly labeled with bromodeoxyuridine by terminal transferase (Roche Molecular Biochemicals) and detected by FITC-conjugated monoclonal antibody to bromodeoxyuridine using the APO-BrdU kit according to manufacturer's protocol (Phoenix Flow Systems, San Diego, CA). Cells were counterstained with 5 microgram/ml propidium iodide containing RNase A for detection of total DNA, and two-color analysis of DNA strand breaks and cell cycle was achieved by flow cytometry. All flow cytometric analyses were performed on an Epics XL Flow Cytometer (Coulter Corp., Miami, FL) equipped with an argon laser. Data was modeled with the Multiplus AV software (Phoenix Flow Systems).

### Preparation of cell lysates, conditioned media and subcellular fractions

For cell lysates, monolayers were washed with PBS, scraped into 2 × Laemlli buffer (containing protease and phosphatase inhibitors) and sonicated prior to protein determination using the BCA protein assay (Pierce, Rockland, IL).

For conditioned media fractions, serum free media from cells was centrifuged (500 × g, 5 min, 4°C) to pellet any floating cells and concentrated using ultra-centrifugation filter units (5 KDa cut-off) according to the manufacturer's protocol (Fisher Scientific).

For subcellular fractions, adherent and floating cells were pelleted by centrifugation (500 × g, 5 min, 4°C), resuspended in wash buffer (25 mM Tris pH 7.5, 250 mM sucrose, 2.5 mM MgCl_2_, protease and phosphatase inhibitors), pelleted (500 × g, 5 min, 4°C), resuspended in 3 volumes of buffer A (20 mM HEPES-KOH, pH 7.5, 10 mM KCL, 1.5 mM MgCl_2_, 1 mM EDTA, 1 mM EGTA, 250 mM sucrose, protease and phosphatase inhibitors), lysed with a Dounce homogenizer, and fractionated by differential centrifugation. Briefly, homogenates were centrifuged twice (500 × g, 5 min, 4°C) and the supernatants transferred to an ultracentrifuge tube. The pellets (containing nuclei) were resuspended in Buffer A, sonicated and stored at -80°C. The combined supernatants were ultracentrifuged (100,000 × g, 1 h, 4°C) to generate the cytosolic fraction (supernatant) and the non-nuclear membrane fraction (NNMF) (pellet). The pellets were resuspended in 100 ml Buffer A, sonicated and stored at -80°C. All protein samples were analyzed for total protein using the Micro BCA protein assay (Pierce, Rockford, IL).

### Western blot analysis

Subcellular fractions, conditioned media, or cell lysates isolated as described above were solubilized in Laemmli sample buffer, separated under reducing conditions by SDS-PAGE and transferred to nitrocellulose. Proteins derived from the NNMF and cytosolic extracts were immunoblotted with a rabbit polyclonal antibody directed against Bax (Clone 13666E; Pharmingen, San Diego, CA) and normalized for protein loading with mouse monoclonal antibodies directed against GAPDH (Clone 6G5; Biogenesis, Brentwood, NH) and ATP synthase (Molecular Probes, Inc., Eugene, OR) diluted 1:500, or 1:1000 respectively. Proteins derived from nuclear fractions were immunoblotted with a mouse monoclonal antibody directed against p21^WAF1 ^(Clone EA10; Calbiochem, San Diego, CA) diluted 1:50. Protein samples were normalized by comparison to a mouse monoclonal antibody antibodies directed against lamin A/C (Clone sc-7293; Santa Cruz, Santa Cruz, CA) diluted 1:100. Proteins derived from cell lysates were immunoblotted with mouse monoclonal directed against ER-alpha (Clone 6F11; Nova Castra, New Castle upon Tyne, UK) or clusterin (Clone 7D1; RDI, Flanders, NJ) diluted 1:50 and 1:1000 respectively and normalized for protein loading by immunoblotting with GAPDH antibody. All antibodies were diluted in PBS plus 5% skim milk. Proteins derived from conditioned media were immunoblotted with anti-clusterin antibody (Clone 7D1; RDI, Flanders, NJ). Specific antibody binding was detected by the appropriate horseradish peroxidase-conjugated secondary antibodies (BioRad, Hercules, CA) diluted 1:5000 in PBS plus 5% skimmed milk and detected using enhanced chemiluminescence. (Pierce, Rockford, IL). The band intensities were measured and analyzed with the Kodak 1D imaging software. Changes in protein levels were normalized relative to the appropriate loading control (lamin A/C for the nuclear fraction; GAPDH for the cytosolic fraction, and ATP-synthase-alpha for the mitochondrial fraction) and then plotted relative to the appropriate control (which was normalized to a value of 1).

### Matrigel invasion assay

Invasion was assessed using 8 micron invasion chambers coated with growth factor reduced Matrigel™ (Becton Dickinson, Bedford, MA) according to the manufacturer's protocol with the exception that fibroblast conditioned media (from NIH 3T3 cells) was used as a chemo-attractant. The number of invasive MCF-7, MCF-7CLU and SUM-159PT cells was determined 24 h after 1 × 10^4 ^cells were seeded into the Matrigel™ chambers. At that time the Matrigel™ was removed from the inserts and the cells on the underside of the membranes were fixed using 1% gluteraldehyde for 15 min, stained with 0.1% crystal violet for 30 min and rinsed with water to remove unbound dye. Membranes were dried, and numbers of invasive cells on each insert were counted by viewing under an Olympus AX70 microscope.

### Orthotopic xenograft of MCF-7CLU cells

Six week old ovariectomized female NCr-*nu *mice (Taconic Farms, Germantown, NY) supplemented with 17-beta-estradiol-sustained release pellets (1.7 mg/90 days) (Innovative Research, Sarasota, FL), were used as hosts for MCF-7 and MCF-7CLU xenografts. Mice were inoculated orthotopically with 2 × 10^6 ^cells/0.1 ml injection directly into the inguinal mammary fat pad under anesthesia. Body weight and tumor size was monitored weekly by caliper measurements of the length, width and height, and volume was calculated using the formula for a semi-ellipsoid (4/3Br^3^/2). When tumor volume reached approximately 200 mm^3 ^(4-5 weeks), mice were randomized to control or tamoxifen treatment groups. Anesthetized mice were implanted subcutaneously with placebo or tamoxifen (15 mg/60 day) continuous release pellets. After 6 weeks of treatment, mice were euthanized, blood was collected by cardiac puncture, tumors and organs were removed, examined and fixed in 4% formalin (Fisher Scientific) for histological analysis. All animal studies were carried out in accordance with an IACUC-approved protocol at the University of Notre Dame.

### Histological analysis of tumors and organs

For routine morphological assessment, tumors and organs were embedded in paraffin, sectioned at 5 microns, and stained with hematoxylin (Gill's formulation 3, Fisher Scientific) and eosin Y (Sigma, St. Louis, MO). Mitotic and apoptotic cells were identified by immunohistochemistry for proliferating cell nuclear antigen (PCNA) and by TUNEL assay. For PCNA, sections were incubated with mouse monoclonal anti-PCNA (Nova Castra Laboratories) at a 1:50 dilution in 1% BSA-PBS. The secondary biotin conjugated antibody (Vector Laboratories, Burlingame, CA) was applied at a 1:200 dilution in 1% BSA-PBS. The ABC technique followed by diaminobenzidine was used to localize peroxidase, and sections were counterstained with Harris' modified hematoxylin (Fisher). TUNEL was performed with a commercially available assay according to manufacturer's directions (Boehringer Mannheim, Indianapolis, IN).

For quantitation of proliferation and apoptosis PCNA and TUNEL positive cells were counted as a percentage of total cells. At least 4 mice from each group were chosen and 2 tumor sections (approximately 500 cells/section) from each mouse were quantitated for PCNA and TUNEL positivity.

### Statistical analyses

Statistical comparisons were performed using Student's unpaired t tests (for two groups) or one-way non-parametric ANOVA for more than two groups. Data are expressed as the mean ± SD or mean ± SEM, depending on the context as indicated in the figure legends, and differences between means were considered significant at P < 0.05.

## Results

**Figure 1:**
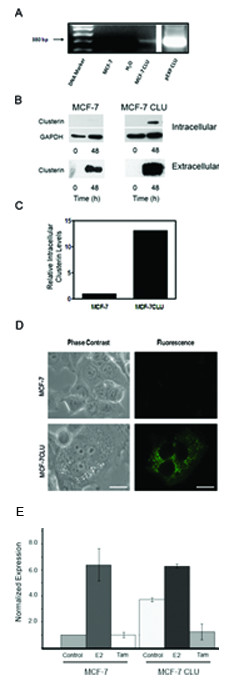
**Over-expression of clusterin in MCF-7 cells**. **Panel A**: Stable transfection of clusterin into MCF-7 cells using the Gateway pDEST-clusterin, verified by PCR amplification. Following stable transfection into MCF-7 cells using the pDEST-CLU generated by the Gateway technology, nuclear DNA was prepared from MCF-7 and MCF-7CLU cells and analyzed for plasmid integration by PCR amplification using clusterin and vector DNA sequences derived from the clusterin cDNA and the vector. The pEXP-clusterin was used as a positive control. **Panel B**: Cell lysates (30 micrograms) and conditioned medium (50 microliters) prepared from MCF-7 and MCF-7CLU before and after 48 h of incubation in serum free medium were separated on 12.5% SDS-PAGE gels and immunoblotted with mouse monoclonal antibodies against clusterin and GAPDH. Blots are representative of three independent experiments. **Panel C**: Comparison of baseline expression of the intracellular clusterin protein expression in MCF-7 and MCF-7CLU cells were normalized against GAPDH. **Panel D**: Localization of GFP-tagged clusterin in stably transfected MCF-7 cells. MCF-7 and MCF-7CLU cells were seeded onto poly-L-lysine coated chamber slides and grown for 48 h prior to fixing and photography. Scale bar: 25 micrometers. **Panel E**: Relative expression of pS2 in MCF-7 and MCF-7CLU cells in response to estradiol. MCF-7 and MCF-7CLU cells were grown in the presence of absence of estradiol and/or 10 micromolar tamoxifen for 48 h. RNA was extracted and the level of relative pS2 mRNA was determined by RT-PCR.

Stable transfection of MCF-7 cells with the pEXP-CLU using the Gateway expression system resulted in the integration of approximately 1-2 copies per cell, giving rise after selection to an MCF-7 cell line constitutively expressing secretory clusterin. This cell line was designated MCF-7CLU (Figure 1 panel A). One drawback of the Gateway system is that it is not possible to produce a companion empty vector control. We have therefore used an untransfected MCF-7 cell line cultured in parallel as the control for these experiments. The intracellular levels of clusterin in the MCF-7 CLU cell line is increased between 10-15 fold by 48 h compared to the parental line (Figure 1 panels B and C). This is reflected by a substantial increase in the levels of extracellular clusterin (Figure 1 panel B), however since it is not possible to normalize the levels of extracellular clusterin, it is only possible to state that the transfected cell line secretes approximately 10-15 times more clusterin than the untransfected, unstressed MCF-7 cells. It is also important to recognize that parental MCF-7 cells also secrete measurable levels of clusterin over the 48 h collection time. The chimeric GFP tagged clusterin protein is excluded from the nucleus and is localized within the endomembrane system, as indicated by the punctate fluorescence, rather than the diffuse staining typical of cytoplasmic localization (Figure 1 panel D). MCF-7 cells over-expressing clusterin retain their sensitivity to both estrogens and anti-estrogens as assessed by monitoring the changes in the steady state level of the estrogen target gene, pS2, by real time PCR (Figure 1 panel E). While the basal level of pS2 mRNA is elevated in untreated MCF-7CLU cells relative to the parental cell line, the estrogen induced expression of pS2 mRNA, and the responsiveness to tamoxifen is essentially equivalent in the parental and MCF-7CLU cell lines, indicating that clusterin over expression has not significantly altered the ability of the estrogen receptor to transactivate known target genes.

**Figure 2:**
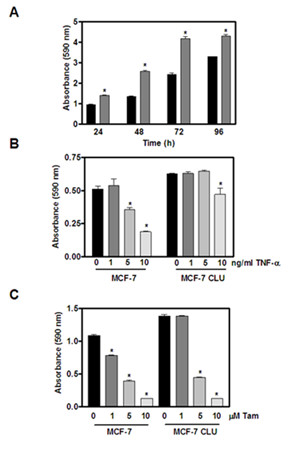
**Effect of TNFalpha and tamoxifen on MCF-7 and MCF-7CLU cell adherent cell number**. **Panel A**: MCF-7 (*black bars*) and MCF-7CLU (*grey bars*) cells were plated at 20,000 cells per well and allowed to grow for the indicated times. Total adherent cell number was determined by crystal violet staining. **Panel B**: MCF-7 and MCF-7CLU cells were plated at 10,000 cells per well. Two days later cells were treated with ethanol vehicle or varying concentrations of TNFalpha (1, 5 and 10 ng/ml) for 48 h. **Panel C**: MCF-7 and MCF-7CLU cells were plated at 10,000 cells per well. Two days later cells were treated with ethanol vehicle or varying concentrations of tamoxifen (1, 5 and 10 micromolar) for 72 h. Data represents mean ± SEM of four values per time point. Similar results were obtained from at least three other independent experiments. *P < 0.05.

MCF-7CLU cells grow more rapidly than untransfected MCF-7 cells (Figure 2 panel A). Flow cytometry demonstrates that this increase in cell number is not associated with dramatic changes in cell cycle (Table 1) but is associated with a significant decrease in apoptosis in MCF-7CLU compared to the parental MCF-7 cells (Table 2). In untransfected MCF-7 cells, TNFalpha induces a decrease in the proportion of cells in S phase with a concomitant increase in the proportion of cells in G_0_/G_1 _(Table 1) as well as an increase in the percentage of apoptotic cells (Table 2). Stable transfection of secretory clusterin abrogates the effects of TNFalpha and tamoxifen on cell cycle kinetics and apoptosis in MCF-7CLU cells, resulting in the overall increase in cell number (Figure 2 panel A). Clusterin over-expression confers significant resistance to TNFalpha, and partial resistance to tamoxifen (since MCF-7CLU cells appear to show resistance to 1 micromolar tamoxifen (Figures 2 panels B and C).

**Table 1:** Effects of TNFalpha and Tamoxifen on MCF-7 and MCF-7CLU cell cycle kinetics.

	G_0_/G_1_		S		G_2_/M	
	MCF-7	MCF-7CLU	MCF-7	MCF-7CLU	MCF-7	MCF-7CLU
Control	61.6 ± 2.8	52.8 ± 2.5*	27 ± 2.1	35.2 ± 4.0*	12.4 ± 1.8	11.5 ± 3.8
Tamoxifen (5 micromolar)	75.2 ± 3.4	60 ± 3.0*	14.5 ± 4.0	31.7 ± 2.1*	9.5 ± 3.0	8.3 ± 1.9
TNF-alpha (5 ng/ml)	72.8 ± 1.4	56.3 ± 2.2*	15.3 ± 3.8	41.9 ± 3.4*	11.9 ± 2.0	1.6 ± 1.2*

MCF-7 and MCF-7 CLU cells were grown in culture and treated with ethanol vehicle, TNFalpha (5 ng/ml) or tamoxifen (5 micromolar) for 48 h. Cells were harvested, ethanol fixed and stained with propidium iodide and analyzed by flow cytometry. Results are expressed as mean ± SD of three independent replicates. (* indicates significant differences between means (MCF-7 versus MCF-7CLU), p < 0.05).

**Table 2:** Effects of TNFalpha and Tamoxifen on MCF-7 and MCF-7CLU DNA fragmentation.

Percentage Apoptotic Cells	MCF-7	MCF-7CLU
Control	13.6 ± 1.4	7.4 ± 1.2*
Tamoxifen (5 micromolar)	22.2 ± 2.8	5.6 ± 1.4*
TNFalpha (5 ng/mL)	22.6 ± 1.8	6.4 ± 1.8*

MCF-7 and MCF-7 CLU cells were grown in culture and treated with ethanol vehicle, TNFalpha (5 ng/ml) or tamoxifen (5 micromolar) for 48 h as described in Materials and Methods. DNA strand breaks were labeled with Br-dUTP and detected using a FITC-conjugated anti-BrdU antibody and analysed by flow cytometry as described in *Materials and Methods*. Data are expressed as the percentage of cells positive for DNA fragmentation. Results are expressed as mean ± SD of three independent replicates. (* indicates significant differences between means (MCF-7 versus MCF-7CLU), p < 0.05).

**Figure 3:**
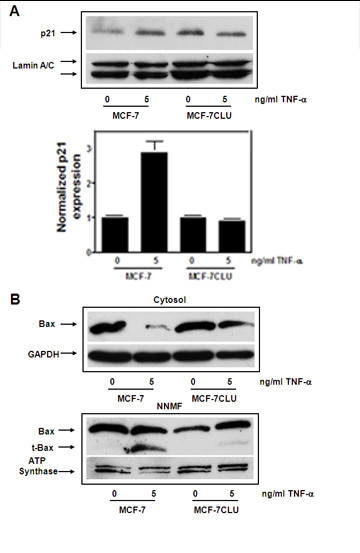
**Effect of TNFalpha on p21 and Bax in MCF-7 and MCF-7CLU cells**. **Panel A**: Nuclear extracts prepared from MCF-7 and MCF-7CLU cells treated with ethanol or 5 ng/ml TNFalpha for 48 h were separated on 10% SDS-PAGE gels and immunoblotted with mouse monoclonal antibodies against p21. Nuclear p21 protein expression was normalized against lamin A/C and plotted relative to appropriate control. Blots are representative of two independent experiments. **Panel B**: Cytosolic fractions prepared from MCF-7 and MCF-7CLU cells treated with ethanol vehicle or 5 ng/ml TNFalpha for 48 h were separated on 12.5% SDS-PAGE gels and immunoblotted with monoclonal antibodies directed against bax and GAPDH. Non-nuclear membrane (NNMF) fractions were separated on 12.5% SDS-PAGE gels and immunoblotted with monoclonal antibodies directed against Bax, GAPDH and ATP synthase. Blots are representative of two independent experiments. Data are expressed as mean ± range.

In untransfected MCF-7 cells, TNFalpha induces the expression of p21 (Figure 3 panel A) and stimulates the proteolytic activation of Bax, an upstream regulatory protein that initiates mitochondrial membrane permeability transition and is surrogate marker for cell death. In contrast in MCF-7 CLU cells, over-expression of secretory clusterin appears to block the effects of TNFalpha on both cell cycle (by blocking the increase in p21) and cell death by preventing the proteolytic activation of Bax (Figure 3 panel B).

**Figure 4:**
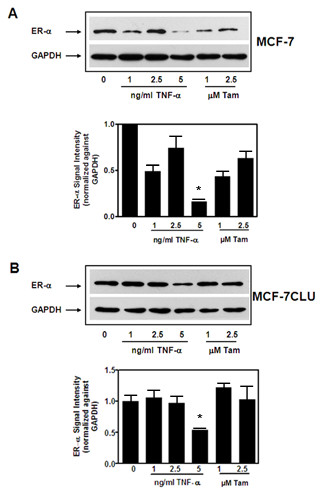
**Effects of TNFalpha and tamoxifen on ERalpha expression in MCF-7 and MCF-7CLU cells**. Total cell lysates prepared from MCF-7 (**Panel A**) and MCF-7CLU (**Panel B**) cells treated with ethanol vehicle, TNFalpha (1, 2.5 and 5 ng/ml) or tamoxifen (1, 2.5 micromolar) in serum free medium for 48 h were separated on 10% SDS-PAGE gels and immunoblotted with mouse monoclonal antibodies against ERalpha and GAPDH. Individual band intensity was normalized against GAPDH and plotted relative to appropriate control. Blots are representative of three independent experiments. Results are expressed as Mean ± SD, *, p < 0.05 (control versus treated).

In the parental MCF-7 cells, TNFalpha induces a dose dependent decrease in ERalpha expression by 48 h (Figure 4 panel A). In contrast, tamoxifen induces a slight increase in ERalpha levels in the untransfected cells. In MCF-7CLU cells the changes in ERalpha expression are substantially reduced but not completely abrogated (Figure 4 panel B).

**Figure 5:**
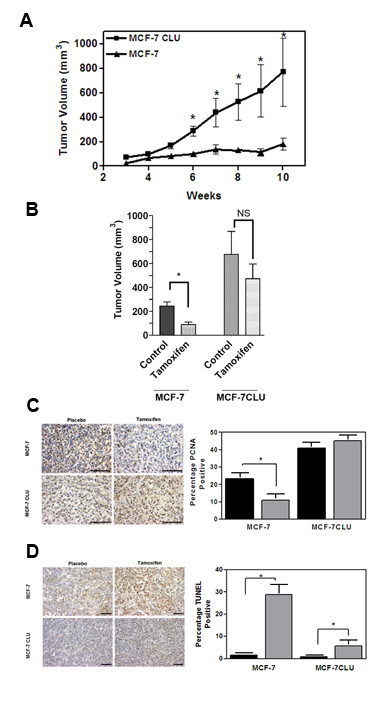
**Response of MCF-7 and MCF-7CLU cells grown as orthotopic xenografts to tamoxifen**. **Panel A**: Ovariectomized nude mice supplemented with slow release estradiol pellets (1.7 mg/90 day) were inoculated with MCF-7 or MCF-7CLU cells and allowed to grow for 4-5 weeks. *P < 0.05; MCF-7 vs MCF-7CLU. (n = 10-12). **Panel B**: Ovariectomized nude mice supplemented with slow release estradiol pellets (1.7 mg/90 day) were inoculated with MCF-7 or MCF-7CLU cells and allowed to grow for 4-5 weeks until tumors measured 200 mm^3^. Animals were implanted with slow release placebo or tamoxifen pellets. Results are expressed as mean ± SD. *P < 0.05 (control versus treated, n = 10). **Panel C**: Formalin-fixed sections from placebo or tamoxifen treated MCF-7 or MCF-7CLU tumors were labeled for cellular proliferation using PCNA. Representative sections stained for PCNA (scale bar: 50 micrometers). Positive cells were counted as a percentage of total cells. A minimum of 500 cells from each of two sections from four mice per treatment group were evaluated. *P < 0.05 control vs treated. **Panel D**: Formalin-fixed sections from placebo or tamoxifen (15 mg/60 day) treated MCF-7 or MCF-7CLU tumors, excised after 10 weeks of treatment, were TUNEL labelled. Representative TUNEL-stained sections (scale bar: 50 micrometers). Positive cells were counted as a percentage of total cells. A minimum of 500 cells from each of two sections from four mice per treatment group were evaluated. *P < 0.05 control vs treated.

**Figure 6:**
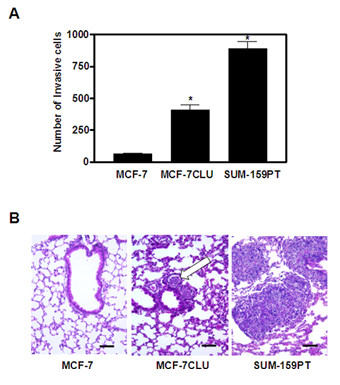
**Invasive and Metastatic Potential of MCF-7 and MCF-7CLU**. **Panel A**: *In vitro *invasive potential was analysed using a Boyden chamber Matrigel™ invasion assay. Cells that invaded through the 8 micron Matrigel™ membrane and attached to the bottom of the well were fixed with 1% gluteraldehyde, stained with crystal violet, allowed to dry and counted using a light microscope. Data represent the mean ± SEM of 3 independent experiments performed in quadruplicate for each cell line. SUM-159PT cells are shown as a positive control. *P < 0.05; MCF-7 vs MCF-7CLU and SUM-159PT. **Panel B**: Representative lung tissue sections from mice inoculated orthotopically with MCF-7 or MCF-7CLU were formalin fixed, paraffin embedded, sectioned and stained with hematoxylin and eosin. Incidence of metastatic lung deposits was monitored in animals 10 weeks after implantation. Arrow indicates MCF-7CLU metastatic foci to the lung. Lung sections from mice inoculated with the metastatic SUM-159PT are shown as positive controls. Bar: 100 microns.

*In vivo*, over-expression of secretory clusterin has a dramatic effect on the growth rate of orthotopic xenografts. As shown in Figure 5 panel A, MCF-7 cells grow slowly, even in estradiol supplemented mice. In contrast, MCF-7CLU cells grow more rapidly, reaching an average volume of 800 mm^3 ^by 10 weeks, although the growth rate of individual tumors is quite variable. Tamoxifen significantly decreases the volume of the tumors derived from untransfected MCF-7 cells, but has no statistically significant effect on the volume of MCF-7CLU tumors (Figure 5, panel B). This is reflected in the decrease in the proportion of replicating cells in tumors derived from untransfected MCF-7 cells compared to MCF-7CLU cells (Figure 5 panel C), and the relative increasing rate of apoptosis in the MCF-7 tumors after treatment with tamoxifen compared to the MCF-7CLU tumors which show a small, but significant increase in the percentage of apoptotic cells (Figure 5 panel D). Over-expression of secretory clusterin also increases the invasiveness of the transfected cells *in vitro *(Figure 6 panel A), and a significant number of primary tumors displayed metastatic progression to the lungs and lymph nodes *in vivo *(Figure 6 panel B and Table 3).

**Table 3:** Frequency of macroscopic metastases in nude mice after orthotopic implantation of MCF-7 or MCF-7CLU cells in the inguinal fat pad of female NCR/nu mice.

	Gross Morphology		
Tumor Type	Lymph Nodes	Lung	Liver
MCF-7	0/35	0/35	0/35

MCF-7CLU	26/35	11/35	1/35

## Discussion

The data presented here demonstrate that over-expression of secretory clusterin alters the phenotypic behavior of MCF-7 breast cancer cells. The decrease in the proportion of MCF-7CLU cells undergoing apoptosis, without a concomitant change in the rate of proliferation results in a more rapid increase in cell number even in unstressed cells. MCF-7CLU cells are also significantly less sensitive to either the anti-estrogen, tamoxifen, or the cytokine, TNFalpha, both of which are known to induce apoptosis in the parental cell line. The reduced sensitivity of the MCF-7CLU to these biological modifiers is manifested by a decrease in apoptotics staining and the abrogation of their effects on proliferation (as measured by ApoBrdU and PI staining respectively) compared to the untransfected parental cell. These effects are mirrored by the abrogation of the increase in p21 levels in the nucleus and the lack of Bax cleavage in response to TNFalpha. There are two possible explanations for these effects. First, as has been suggested from previous studies, clusterin may bind to TNFalpha in the medium [23,24], substantially reducing the effective concentration of TNFalpha. Alternatively, in MCF-7CLU cells treated with TNFalpha or tamoxifen, the biogenesis of the newly synthesized clusterin may be modified, altering the intracellular signaling pathways responsible for the induction of cell cycle arrest and apoptosis. Two different mechanisms have been proposed to account for the changes in clusterin biogenesis. It has been suggested that under stress conditions the splicing of the clusterin primary transcript is altered, producing a mature mRNA that is translated on free ribosomes from an alternative start site resulting in a truncated protein that can be translocated to the nucleus [35]. Over-expression of this clusterin isoform, which binds to Ku70/80 has been shown to induce apoptosis in MCF-7 cells [13]. In the current studies, we have not been able to identify the variant RNA using RT-PCR, nor have we identified a protein of the appropriate molecular mass (49 kDa) by Western analysis (data not shown). Our previous studies have suggested that in MCF-7 cells clusterin biogenesis is altered by profound changes in the post-translational modifications that occur after treatment with tamoxifen. These changes appear to occur after the full length mRNA has been translated by bound ribosomes [12]. During the process of co-translational translocation through the translocons of the endoplasmic reticulum, nascent clusterin is normally glycosylated by oligosaccharyltransferase a process that is regulated in part by Ost2p (DAD-1, defender against death-1) [36,37]. The glycoprotein is then further modified and disulfide bonded in the ER, translocated to the Golgi, and cleaved to form the mature alpha and beta disulfide linked chains in the trans Golgi [12]. Over-expression of clusterin or other secretory proteins does not appear to induce an ER-overload response [38], and it is unlikely that the glycosylation, processing or trafficking of the glycoprotein is altered as a consequence of over-expression. In MCF-7 cells, during apoptosis induced by TNFalpha or anti-estrogen treatment, clusterin expression is significantly upregulated [12]. However, Ost2p/DAD-1 is also inactivated [39,40] and the initial glycosylation of clusterin (and other glycoproteins) is abrogated as the structural integrity of the oligosaccharyltransferase is compromised [41]. While this does not interfere with the translation or translocation of the nascent protein, or its subsequent folding and disulfide bond formation, the non-glycosylated protein is not cleaved into the mature heterodimer in the Golgi. This induces ER overload [38] resulting in the appearance of the uncleaved, disulfide-linked protein in the nucleus of dying cells [12]. The retro-translocation of clusterin from the endomembrane system to the cytoplasm has recently been confirmed [42], providing an important missing component in the biosynthesis of the disulfide linked nuclear isoform. These latter studies also provide a rational explanation for the presence of the protein in the cytoplasmic compartment where it has been shown to influence TGFbeta-mediated signaling and inhibit apoptosis through a direct interaction with activated Bax [43].

The observation that clusterin over-expression also stimulates invasion and metastatic progression, at least to the lungs, suggests that the secreted glycoprotein has additional extracellular functions. This increase in the number of metastatic sites does not appear to correlate simply with the increased size of the MCF-7CLU primary tumors, although we cannot definitively rule out this possibility. In our view it is more likely that secretory clusterin plays one or more distinct roles in the metastatic spread of the cells from the primary tumor. Hematogeneous spread of tumor cells is a complex process that involves intravasation into the microvasculature surrounding the primary tumor, survival of tumor cells in the circulation and extravasation from the microvasculature at the metastatic site [44,45]. For many metastatic cells, survival is enhanced through heterotypic cell-cell interactions, which promotes embolus formation. Embolus formation also increases the likelihood that invasive cells arrest in the microvasculature at the metastatic site, and enhances the probability that the cells will extravasate. It is likely that chronic over-expression of clusterin in cells which survive therapy results in the clustering of tumor cells, platelets and other circulating cells to form emboli, possibly as a by-product of the function of the glycoprotein as an extracellular chaperone, as originally described by Fritz and his colleagues who first described and named the protein [46].

While the experimental design used in this study cannot exclude the possibility that some of the clusterin synthesized within the endomembrane system is retro-translocated to the nucleus even in the unstressed cells over-expressing the secretory isoform, we were unable to detect any nuclear clusterin prior to treatment with TNFalpha or tamoxifen (Figure 1D and data not shown). This suggests that the phenotypic changes seen in the unstressed MCF-7CLU cells are probably due to the secretory glycoprotein rather than intracellular isoforms. However, it is likely that changes in endomembrane trafficking accompanying the response of the MCF-7CLU cells to TNFalpha (*in vitro*) and tamoxifen (*in vivo*) result in ER overload and the retrotranslocation of a proportion of the nascent protein to the nucleus, although we have been unable to detect the nuclear isoform during treatment with tamoxifen or TNFalpha or in surviving cells using immunofluorescence. Thus, it is not possible to fully delineate which of the observed effects on the phenotype of the MCF-7CLU after treatment are due to effects of the secretory isoform and which are due to the cytoplasmic or nuclear isoforms. In fact, it is probable that the phenotypic changes in the MCF-7CLU cells, including their increased resistance to cell death and the acquisition of the invasive phenotype represents the combined effect of the extracellular and intracellular isoforms. Further work will be required to dissect these responses and refine the target for interventional therapies such as the anti-sense approaches currently being pursued by others for the treatment of prostate cancer.

## Conclusions

These data demonstrate that secretory clusterin, which is frequently up-regulated in breast cancers by common therapies, including anti-estrogens, may play a significant role in tumor growth and metastatic progression by blocking the apoptotic signaling, leading to cell survival and by increasing the ability of cells to survive during one or more phases of the metastatic process. Targeting the secreted form of clusterin may decrease the rate of metastatic progression.

## Competing interests

The authors declare that they have no competing interests.

## Authors' contributions

LF was responsible for the *in vivo *studies described in this manuscript. LW was responsible for the *in vitro *experiments, including the cloning and over expression of clusterin. NC performed the experiments designed to demonstrate that the over-expression of clusterin did not alter the sensitivity of the transfected cells to estradiol. MT conceived and designed the experiments described in this manuscript. All authors collaborated to analyze the data, and write the manuscript.

## Pre-publication history

The pre-publication history for this paper can be accessed here:

http://www.biomedcentral.com/1471-2407/10/107/prepub
